# Neonatal circulatory failure due to acute hypertensive crisis: clinical and echocardiographic clues

**DOI:** 10.5830/CVJA-2013-003

**Published:** 2013-04

**Authors:** Jacoba Louw, Stephen Brown, Benedicte Eyskens, Ruth Heying, Bjorn Cools, Marc Gewillig, Liesbeth Thewissen, Karel Allegaert, Anne Smits, Elena Levtchenko

**Affiliations:** Paediatric Cardiology, University Hospitals Leuven, Leuven, Belgium; Paediatric Cardiology, University Hospitals Leuven, Leuven, Belgium; University of the Free State, Bloemfontein, South Africa; Paediatric Cardiology, University Hospitals Leuven, Leuven, Belgium; Paediatric Cardiology, University Hospitals Leuven, Leuven, Belgium; Paediatric Cardiology, University Hospitals Leuven, Leuven, Belgium; Paediatric Cardiology, University Hospitals Leuven, Leuven, Belgium; Neonatology, University Hospitals Leuven, Leuven, Belgium; Neonatology, University Hospitals Leuven, Leuven, Belgium; Paediatric Nephrology, University Hospitals Leuven, Leuven, Belgium; Paediatric Nephrology, University Hospitals Leuven, Leuven, Belgium

**Keywords:** neonatal, shock, hypertension, crisis, echocardiography

## Abstract

**Objective:**

Circulatory failure due to acute arterial hypertension in the neonatal period is rare. This study was undertaken to assess the clinical and echocardiographic manifestations of circulatory failure resulting from acute neonatal hypertensive crisis.

**Methods:**

Neonatal and cardiology databases from 2007 to 2010 were reviewed. An established diagnosis of circulatory failure due to neonatal hypertension before the age of 14 days was required for inclusion. Six patients were identified.

**Results:**

Five patients presented with circulatory failure due to an acute hypertensive crisis. The median age at presentation was 8.5 days (range: 6.0–11.0) with a median body weight of 3.58 kg (range: 0.86–4.70). Echocardiography demonstrated mild left ventricular dysfunction [median shortening fraction (SF) 25%, range 10–30) and mild aortic regurgitation in 83% (5/6) of patients. One patient with left ventricular dysfunction (SF = 17%) had a large apical thrombus. Two patients were hypotensive, and hypertension only became evident after restoration of cardiac output. Administration of intravenous milrinone was successful, with rapid improvement of the clinical condition. Left ventricular function normalised in all survivors.

**Conclusion:**

Early neonatal circulatory collapse due to arterial hypertension is a rare but potentially life-threatening condition. At presentation, hypotension, especially in the presence of a dysfunctional left ventricle, does not exclude a hypertensive crisis being the cause of circulatory failure. The echocardiographic presence of mild aortic regurgitation combined with left ventricular hypocontractility in a structurally normal heart should alert the physician to the presence of underlying hypertension.

## Abstract

Circulatory failure is frequently the forerunner of a serious underlying condition in the neonate. It requires rapid detection of origin to differentiate non-cardiac (e.g. sepsis, metabolic derangements) from cardiac causes. Echocardiography is requested as an early diagnostic test, primarily with the intention to exclude potentially lethal structural heart disease, for example coarctation of the aorta, critical aortic stenosis, or hypoplastic left heart. When left ventricular dysfunction is observed, the focus shifts to myocardial disease. Acute episodes of systemic hypertension are rarely, if ever, considered in the differential diagnosis.

Very little is known about the incidence of acute hypertensive crisis in newborn infants. Acute systemic hypertensive episodes during this period are most likely under-recognised and underdiagnosed. It is a rare but potentially life-threatening condition. Systemic hypertension in neonates has a reported incidence varying from 0.2 to 2.6%.^1^-^4^

Cardiomegaly, hypocontractility, overt cardiac failure and even death related to neonatal hypertension have been described but consist mostly of a few case reports.^2^,^3^,^5^-^10^ Successful management relies on early and prompt recognition and treatment.

This study was undertaken to assess the clinical and echocardiographic manifestations of circulatory failure resulting from acute hypertensive events.

## Methods

This was a retrospective review. To be considered for inclusion, an established diagnosis of circulatory failure associated with a blood pressure more than the 95th percentile for gestational age and weight during the course of admission in the neonatal intensive care unit before the age of 14 days was essential.^3^ Neonatal and cardiology databases of a tertiary referral centre from 2007 to 2010, consisting of 2 632 admissions to the neonatal intensive care unit, were reviewed. Six patients with circulatory collapse and documented systemic hypertension, as defined, were identified.

Standard descriptive and demographic data were obtained from patient records. Patient charts were reviewed for clinical course, laboratory results and outcome. In order to eliminate inter-observer bias, echocardiograms performed during the initial presentation were digitally reviewed by a single paediatric cardiologist and recalculated. The averages of previous and recalculated measurements were used.

Left ventricular systolic function, end-diastolic dimension and wall thickness were measured by means of standard M-mode and two-dimensional views. Using previously published normal values, *z*-scores were calculated.^11^,^12^ Mitral and aortic regurgitation were classified according to width and length of colour Doppler jet as mild (jet < 25%), moderate (jet 25–50%) or severe (jet > 50%).

Approval by the local medical ethics committee was obtained. Data were analysed using standard statistical software (SPSS for windows®, Chicago, Illinois, USA, version 18). Data are presented as medians with minimum and maximum values where appropriate.

## Results

The median age at presentation was 8.5 days (range: 6.0–11.0) with a median body weight of 3.58 kg (range: 0.86–4.70). All infants were full term, except one 28 weeks premature infant with a birth weight of 860 g. All patients had circulatory failure and required admission to the intensive care unit for cardiopulmonary support. At initial presentation, three infants had hypertension while two patients were hypotensive and one normotensive. Hypertension in the latter only became evident after resuscitation and restoration of cardiac output. Common symptoms included lethargy, feeding intolerance or poor feeding. Patient characteristics are depicted in Table 1.

**Table 1:** Clinical Characteristics At Presentation

						*Echocardiography*			
	*Weight (g)*	*BP (mmHg)*	*Lactate (mmol/l)*	*Associated symptoms*	*Central line*	*Regurgitation*	*SF %*	*Findings*	*Anti-HT Rx follow up*
1	3210	–	2.5	shock, respiratory distress	–	AR, MR	10	–	yes
2	4700	160/110	1.4	RF	umbillical, venous	AR, MR	30	–	no
3	3650	–	3.2	shock	umbillical, arterial and venous	AR, MR	17	vascular insult right kidney	no
4	3520	140/110	2	feeding intolerance, electrolyte disturbances	femoral, venous	MR	25	–	no
5	3680	100/70	11	distended abdomen	femoral, venous	AR, MR	25	dysplastic right kidney	yes
6	860	–	1.7	poor circulation	umbillical, arterial and venous	AR, MR	poor*	thrombus occlusion descending aorta	died

BP: blood pressure at initial presentation, SF: shortening fraction at initial presentation, R: respiratory failure, AR: aortic regurgitation, MR: mitral regurgitation, R: right, anti-HT Rx: antihypertensive medication. *Extremely poor contractility on visual inspection, measurements not possible (initial echocardiogram).

Umbilical arterial lines had been inserted in two children prior to the episodes of hypertension. None of the mothers were diabetic and maternal health was good except in one where a history of herpes infection and pre-eclampsia was present. There was no history of ingestion of drugs associated with hypertension.

Lactate levels were elevated in most infants on admission (Table 1), with a median of 2.6 mmol/l (range: 1.4–11). Troponin levels were measured in three patients and were elevated in patients 1 and 5: 0.16 and 2.01 µg/l, respectively (normal: < 0.13 µg/l). Both plasma renin activity (PRA) and aldosterone levels were markedly elevated in five patients, with medians of 32.6 µg/l/h (range 10.5–> 37) (local laboratory neonatal reference value: < 16.6 µg/l/h) and 2 396 ng/l (range: 763–24 920) (local laboratory neonatal reference value: 7–184), respectively. In one patient aldosterone levels were within the normal range, but PRA was elevated (37.4 µg/l/h). Urine catecholamines were normal in all patients. Plasma PRA and aldosterone levels gradually decreased and normalised in all patients after a median of eight days (range: 7–13) and antihypertensive treatment could be diminished or discontinued.

Systolic cardiac function was considerably impaired in three patients and mildly impaired in two, with a median SF of 25% for the group as a whole (range: 10–30%, Table 1), while left ventricular end-diastolic dimensions were within normal reference ranges (Table 2). Left ventricular posterior wall thickness was increased in three and interventricular septal thickness in four patients, respectively (Table 2).

**Table 2:** Echocardiographic Findings

	*LVEDD*	*IVS*	*LVPW*
Measurement (mm)			
median	19	5	5
minimum	16	4	4
maximum	21	10	5
z-score			
median	1.1	4.2	2.5
minimum	–0.6	0.8	0.2
maximum	2	5.9	2.5

LVEDD: left ventricular end-diastolic diameter, IVS: interventricular septum, LVPW: left ventricular posterior wall.

**Fig. 1.**
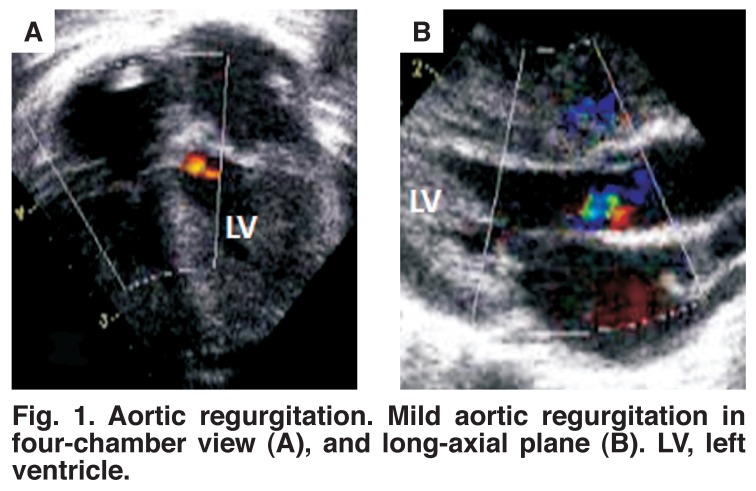
Aortic regurgitation. Mild aortic regurgitation in four-chamber view (A), and long-axial plane (B). LV, left ventricle.

**Fig. 2.**
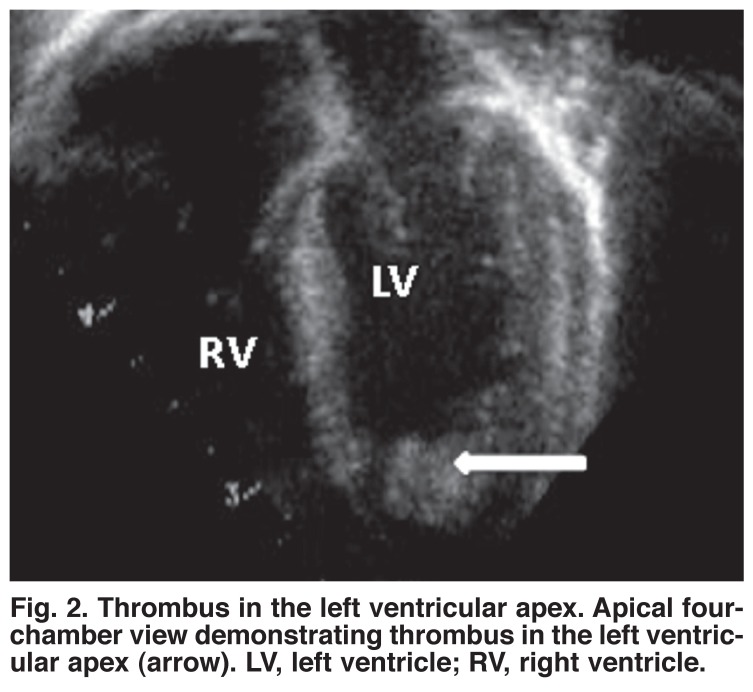
Thrombus in the left ventricular apex. Apical fourchamber view demonstrating thrombus in the left ventricular apex (arrow). LV, left ventricle; RV , right ventricle.

**Fig. 3.**
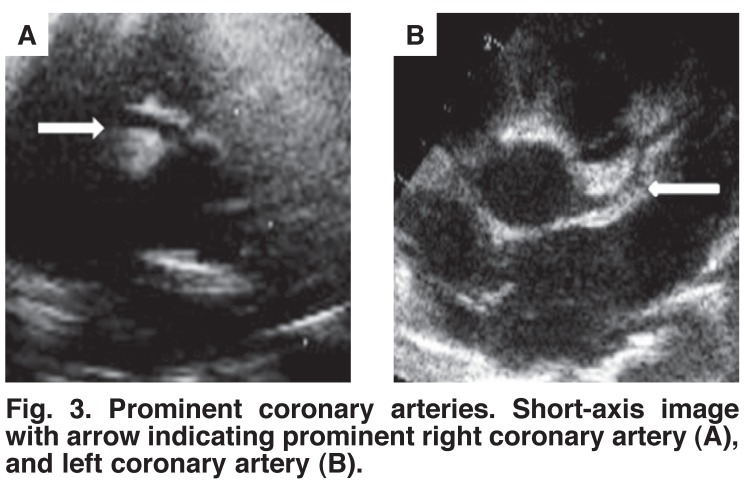
Prominent coronary arteries. Short-axis image with arrow indicating prominent right coronary artery (A), and left coronary artery (B).

In one patient with considerable hypocontractility, a thrombus of 5 × 9 mm was observed in the apex of the left ventricle (Fig. 1). Mild to moderate aortic regurgitation was seen in five of the six patients (Fig. 2) and mild to moderate mitral regurgitation (MR) in all (Table 1). All aortic and mitral valves were morphologically normal. Interestingly, the coronary arteries were reported to be more prominent than usual in four patients on visual inspection of the echocardiograms (Fig. 3).

Milrinone was used in five patients to treat circulatory failure. All patients improved rapidly with normalisation of left ventricular systolic function. The majority of patients required at least two antihypertensive drugs to control the blood pressure in the intensive care unit once they were stabilised.

Underlying causes of hypertension were found in three patients. One patient had a vascular insult of the right kidney and one a dysplastic right kidney. Thrombosis of the abdominal aorta was seen in the 860-g neonate who did not survive. Both this patient as well as the infant with vascular damage of the kidney had umbilical arterial lines prior to presentation. No demonstrable underlying cause could be identified in the other three patients.

Four patients are currently being followed up, with ages of 0.7, 1.0, 3.0 and 3.7 years, respectively. In three of these, permanent renal sequelae are present, namely renal atrophy, dysplastic kidneys, asymmetric renal function and proteinuria. In one of these, cortical atrophy with dysfunction of the kidney only became apparent during follow up. Two still require antihypertensive medication, but all have a normal shortening fraction.

## Discussion

Over a four-year period in a busy neonatal unit in a referral centre, only six patients with circulatory failure due to acute hypertensive crisis were identified, indicating how rare this condition is. These patients are usually critically ill and may die if not diagnosed and treated promptly.

All our patients presented within two weeks of birth. This, remarkably, resembles findings in the only other previously published series where the median age at presentation was 7.2 days.^13^ Similar to our findings, they also observed that the initial clinical symptoms were mostly non-specific and related to feeding and respiratory difficulties.

It is of clinical significance that all our patients presented with some form of circulatory failure. Although most were hypertensive (*n* = 3), two patients presented with hypotension and shock and one was normotensive (Table 1). Hypertension due to increased systemic vascular resistance only became apparent after they were stabilised and resuscitated. The hypotension was most likely caused by impaired left ventricular systolic performance as confirmed by reduced fractional shortening.

In most of our patients, hypertrophy of the interventricular septum and/or left ventricular posterior wall was evident. This increase in left ventricular mass had also been reported by Peterson.^13^ Hypertension in our patients was most likely of recent postnatal onset. We postulate that antenatal onset of hypertension is unlikely, since one then would have expected significant biventricular hypertrophy with significant pulmonary hypertension, Such patients present with cyanosis due to atrial right-to-left shunt.^14^

The differential diagnosis of neonatal hypertension has been extensively reviewed.^3^ An important question to be answered is what triggers these postnatal arterial hypertensive events? Could it be related to the postnatal haemodynamic and humoral changes which ‘relax’ the homeostatic vasomotor tone and elicit an acute biochemical response? Alternatively, is it due to mostly intrinsic renal abnormalities which then become manifest, or are these events triggered by iatrogenic factors such as thrombi from umbilical lines? Thromboembolic events related to umbilical lines are acknowledged as the most common cause of clinical hypertension in neonates. In this study, renal causes were identified in two infants and thrombus in one. More studies are needed to answer these questions.

Echocardiography is usually requested once an infant with circulatory failure is admitted to the neonatal intensive care unit. Faced with this clinical presentation, the demonstration of hypocontractility would inevitably lead the cardiologist to consider a differential diagnosis of myocarditis, cardiomyopathy, coarctation of the aorta or coronary artery anomalies. Left ventricular hypocontractility in the absence of hypertension will be misleading in this case. However, careful analysis of the above mentioned echocardiographic findings associated with mild aortic regurgitation should alert the physician to consider systemic hypertension as the probable underlying cause.

Mitral regurgitation is not unexpected in the presence of left ventricular dysfunction and is frequently observed in association with cardiomyopathy. However, aortic regurgitation is very unusual in a supposedly normal heart, provided the valve is structurally normal. Although the left heart was not dilated, central aortic and mitral valve regurgitation were seen in the majority of patients. We did not measure aortic diameter in this study but Peterson and coworkers reported mild dilation in their study, which they ascribed to the increased aortic distensability in neonates.^13^,^15^ This could possibly provide an explanation for the mild to moderate AR observed in our patients.

Left ventricular hypocontractility was observed in a significant number of our patients. Acute decompensation can be explained on the basis of normal neonatal cardiac physiology. The neonatal heart has, by way of its structure, function and unique changes in loading conditions, limited cardiac reserves and therefore also less compensatory ability. After birth, volume load on the left heart increases sharply, from less than 35% of the total output to 50% of combined ventricular output. Afterload also increases due to marked increase in the systemic vascular resistance. If an additional insult such as the acute pressure load of arterial hypertension is added to an ‘untrained’, adapting left ventricle, acute left ventricular failure becomes a realistic scenario.

The dilated coronary arteries in two-thirds of our patients are intriguing. Adult studies have shown coronary arteries to proportionately increase in diameter in association with left ventricular hypertrophy.^16^,^17^ Furthermore, coronary arteries rapidly dilate in response to increased myocardial demand.^18^ Taken as a constellation of findings, one can therefore deduce that the combination of all these echocardiographic findings would indicate acute cardiac overload of recent onset.

Cardiac failure was treated using milrinone, which in our clinical judgment gave a beneficial response in all our patients and improved cardiac function. Milrinone is a treatment of choice under these circumstances since the phosphodiesterase inhibitors decrease systemic and pulmonary vascular resistances and increase cardiac contractility.^19^

It should be noted that the reference ranges for plasma renin activity show marked variation and vary from laboratory to laboratory. Although transient elevation of PRA or/and plasma aldosterone levels, with subsequent normalisation after control of hypertension, was observed in all patients, underlying renal causes could only be demonstrated in two patients during admission. Whether this renin–angiotensin–aldosterone system activation was part of a reactive stress response or a causative event remains unclear.^18^,^20^,^21^ Nevertheless, outcome in general was good, with gradual disappearance of hypertension and the normalisation of PRA and aldosterone levels in all patients.

## Limitations

The study was limited by the small number of patients and its retrospective nature. In order to reduce observer bias and variability, all echocardiograms were independently re-examined by a single cardiologist. As a consequence of the small sample of patients, the true incidence of hypertension cannot be determined. Only patients with circulatory failure were included, therefore patients with milder forms of disease may not have been detected. Diastolic functional assessments were not carried out at admission and are therefore not available. Further studies are required to elucidate the underlying pathophysiology.

## Conclusion

Findings of this study are based on a limited number of patients. However, early neonatal circulatory collapse due to arterial hypertension is a rare but potentially life-threatening condition. The echocardiographic presence of mild aortic regurgitation combined with left ventricular hypocontractility in an otherwise structurally normal heart should alert the physician to the possibility of underlying hypertension. Clinicians and echocardiographers should be aware that at presentation, hypotension, especially in the presence of a dysfunctional left ventricle, does not exclude a hypertensive crisis and failure to consider the diagnosis may lead to death. Long-term cardiac outcome in survivors appears good.
